# Experiences of Nursing Instructors Related to Safety Issues Using Students as Practice Models in Laboratories: A Focus Group Study

**DOI:** 10.3390/ijerph192417081

**Published:** 2022-12-19

**Authors:** Youngmi Kang, Dongwon Choi, Soohyun Park

**Affiliations:** 1 College of Nursing Science, East-West Nursing Research Institute, Kyung Hee University, Seoul 02447, Republic of Korea; 2 College of Nursing, Incheon Catholic University, Incheon 21987, Republic of Korea; 3 Department of Nursing, Eulji University (Seongnam), Seongnam-si 13135, Republic of Korea

**Keywords:** nursing, faculty, laboratories, students, safety

## Abstract

Aim: Nursing school students perform invasive (i.e., injection, venipuncture) and/or non-invasive procedures (i.e., giving a bed bath and back massage) on each other to master these skills, and nursing instructors reported related safety issues. This study aimed to explore nursing instructors’ experiences concerning their students’ psychological and physical safety when using students as practice models in nursing skills laboratories. Methods: A qualitative design using focus group interviews and thematic analysis was employed. Two semi-structured focus group interviews were conducted with a purposive sample of eight instructors with experience in teaching nursing skills in laboratories. This study was evaluated by the Institutional Review Board at Eulji University (EU18-51) in the Republic of Korea. Results: Three main themes emerged to describe nursing instructors’ safety-related experiences when using students as practice models in nursing skills laboratories: (1) a dilemma between the experimental learning of students and the need to keep students safe, (2) perception related to psychological safety, and (3) an inadequate safety reporting system. Conclusions: When instructors consider using students’ bodies to practice nursing skills, they experience a dilemma between the students’ experimental learning and the need to keep them safe. Thus, methods to maximize student learning and student safety guidelines should be developed.

## 1. Introduction

The effectiveness and quality of preparing undergraduates to enter the nursing profession begin with coursework in the fundamentals of nursing and nursing skills laboratories. One notable pedagogical challenge in teaching nursing skills is encouraging students to translate their theoretical knowledge into clinical practice [1]. Numerous learning methods, including the use of simulators, manikins, and models, have proven effective in improving nursing students’ self-efficacy, self-confidence, and self-perceived competency in nursing skills [1,2,3]. Self-perceived competence refers to *“students’ belief in their own knowledge and practical skills applied to nursing care”* [4]. A nursing student’s self-efficacy, self-confidence, and competence in developing nursing skills are influenced by the learning environment, the teaching modality, as well as the ability to self-reflect [1,3].

Although various methods are frequently used for initial learning, few nursing schools have historically developed nursing skills in a Fundamentals of Nursing laboratory by using students as practice models [5,6]. The use of student-on-student practice continues to be commonplace in nursing programs in the Republic of Korea. Students experience nursing procedures on their body directly: vital signs, incentive spirometry, and a range of motion exercise, position change, hot bag, ice bag, oral medication, blood sugar test, subcutaneous injection, intravenous injection, and intramuscular injection [7,8]. Although there is a potential risk of injury during skills laboratories, empirical evidence regarding the use of students for peers’ practice of procedures during nursing skills laboratories is limited [9].

Similarly, research has been conducted on the incidence of physical injury (especially needle sticks), emotional labor, risk factors, and strategies for ensuring safety among nursing students in clinical settings [10,11]. The lack of information regarding the advantages and disadvantages of such practices for nursing education may lead to problems being overlooked by instructors. Thus, further research is needed to investigate the current state of nursing skills laboratories and assess how practices can be improved, specifically the use of peers as practice models. To our knowledge, no empirical research has explored the pros and cons of the educational method of having students practice procedures on their peers. Moreover, this lack of information prevents nursing educators from completely understanding nursing student safety in nursing skills laboratories.

Not only patient safety, but also nursing students’ safety and rights, are important factors in nursing education. Further investigation to better understand the balance between the risks and benefits of practicing invasively and non-invasively on students’ own bodies is crucial for the effective education programs design. Therefore, the purpose of this study is to examine the experiences of nursing instructors who have taught nursing skills in laboratories regarding the use of student-on-student skills practice, particularly concerning the psychological and physical safety of students, through a focus group study in South Korea. These findings could strengthen the understanding of current classroom methods and problems in nursing skills practice of professionals working in this field, as well as encourage discussion on alternative teaching methods for student-on-student learning. Furthermore, the study will provide insights for establishing legal and institutional safeguards.

## 2. Methods

### 2.1. Design

This study used qualitative content analysis to explore the distress of nursing instructors regarding the psychological and physical safety of the students when using peers as practice models. A qualitative design based on a constructivist paradigm using focus group interviews was employed. This approach was adopted because constructivism aims to investigate the existing variety of constructs and foster a consensus understanding from the perspective of those who are experiencing these constructs [12,13]. This method was chosen to obtain an in-depth understanding of the use of students as practice models in nursing skills laboratories and to provide an opportunity to observe group interactions between purposely selected nursing instructors.

### 2.2. Participants

A purposive sample of eight nursing instructors teaching nursing skills laboratory was recruited to explore their opinions and experiences. They were recruited from the Korean Academy of Fundamentals of Nursing and were enrolled in eight universities throughout the Republic of Korea. The inclusion criterion for participants was having at least three years of teaching experience in nursing skills laboratories. None of the participants refused to participate or dropped out of the study.

Participants’ general characteristics are summarized in Table 1. All participants were women with a mean age of 51.75 and a mean clinical career length of 3.86 years. Their average teaching experience was 17.25 years.

### 2.3. Data Collection

Qualitative data were collected using semi-structured, in-depth focus group interviews with participants on 31 July 2018. The principal investigator prepared the interview guidelines by reviewing the literature on the topic and drawing on her expertise in focus-group research. The guidelines were, in turn, validated by other researchers and revised after receiving their feedback. Prior to conducting the interviews, the general characteristics (age, gender, etc.) were collected. Two interviews, each lasting approximately 90–120 min, were conducted face-to-face by the researchers until data saturation was achieved. All interviews took place in a classroom at University A and were audio recorded. The interviews were transcribed verbatim, and no one other than the researcher and participant was present in the classroom during the interviews.

Several open-ended questions were used to encourage discussion on teaching experiences and perceptions when teaching skills laboratories, for example: “*How do you think about practicing models in nursing skills laboratories, and what specific concern do you have about the students’ practice on each other’s bodies?*”. A second interview was conducted to verify and further explore the participants’ previous statements, which included, for example: “*What is the most difficult aspect that you have experienced in the student practice model? How do you think student practice in laboratories can be improved?*”. These questions were sent to the participants via email prior to the interviews to provide them with an opportunity to reflect on their perceptions. Interview transcripts were created after each interview and were provided via email to participants to confirm that the content accurately represented their meaning and perceptions.

### 2.4. Data Analysis

There are several methods available for analyzing and organizing the qualitative data including mixed method; statistical programs such as ATLAS. Ti; and analytic hierarchy process [14]. However, we used qualitative, inductive content analysis, as suggested by Elo and Kyngäs [15]. This method includes open coding, creating categories, and abstraction. For the open coding process, the first and corresponding authors read the interview transcripts repeatedly to immerse themselves in the data and obtain a sense of the entire text [16]. We strived to avoid any pre-perception of the phenomenon to help new insights emerge throughout the research process [17]. Subsequently, we read the transcripts word for word while highlighting the words that captured the key concepts to derive codes. We then made notes, selected meaningful phrases, and initiated the data analysis. Next, we collected the headings and generated categories and themes.

At the stage of creating the categories and themes, all 3 researchers sorted the lists of the 60 codes under higher-order headings based on their relatedness [15]. At the abstraction stage, we extracted sentences and paragraphs that were meaningful in terms of the research topic, which were then grouped into categories/themes and subcategories/subthemes based on similarity [12]. The first and corresponding authors independently performed the open coding and theme generation process, after which we reached a consensus through discussion [18]. Using this process, we identified three themes and four sub-themes.

### 2.5. Ethical Considerations

This study was conducted with the approval of the Bioethics Review Committee (IRB No. XX18-51). The purpose and procedure of the study were explained to the participants, and they were asked to voluntarily sign a consent form. Participants were assured that any personal information and the interview content would be kept confidential and used only for research purposes. Furthermore, participants were told that they had the right to withdraw at any time and that their participation was voluntary.

### 2.6. Validity and Rigor

To increase the trustworthiness of the results, we used the four evaluation criteria suggested by Lincoln and Guba [19]: credibility, conformability, dependability, and transferability. To increase credibility, we repeatedly read and compared the focus group transcripts. In addition, we contacted the participants and verified the accuracy of the transcript contents. To ensure conformability, we used an established method of content analysis. We maintained reflexivity throughout the research process when developing labels for codes, by reflecting on the prior understanding of practice in skills laboratories [18] to validate the dependability of data collection procedures and data analysis. To ensure transferability, we provided details of descriptive data, such as sample strategy, demographics, inclusion criteria, interview procedure, and questions based on an iterative research process.

## 3. Results

From our findings, three main themes emerged from instructors’ experiences when using students as practice models in nursing skills laboratories: (1) a dilemma between the experimental learning of students and the need to keep them safe, (2) perception related to psychological safety, and (3) an inadequate safety reporting system. The subheadings were developed with one to three items for each theme, in total resulting in six subthemes.

### 3.1. Theme 1. A Dilemma between the Experimental Learning of Students and the Need to Keep Them Safe

Participants mentioned experiencing conflict between ensuring students’ physical safety and promoting learning through peer practice. Instructors mentioned that it is difficult to stop students from practicing nursing procedures on each other due to the virtue of learning experiences. However, they did not deny the possibility of classroom accidents and the fact that instructors need to take responsibility for certain adverse events as major stressors.

#### 3.1.1. Positive Effects from Students Practicing Skills on Each Other

The participants mentioned the positive effects of students practicing skills on each other, such as developing awareness and sensitivity toward patients’ feelings and understanding what it feels like to receive clinical care.

*When comparing the practice of models to the practice of human bodies, satisfaction and confidence were higher*.

(Participant A)

*There’s so much difference between what you do in manikin models and what you do in a real person… Due to the experience of practicing skills on each other, the student said that she was able to adapt to the clinic*.

(Participant H)

#### 3.1.2. Negative Effects from Students Practicing Skills on Each Other

Participants complained of dilemmas resulting from the risks to students’ physical safety in nursing skills laboratories and the need to keep students safe by not performing unnecessary clinical tasks on them. Instructors also mentioned that one of the most challenging aspects of managing classroom safety was avoiding physical risks to students. Common risks to students’ physical safety include puncture wounds from needles, ampoule fragments, and bruising after intravenous injection practice. However, because of the low quality of available manikins and insufficient practice materials, several instructors have few alternative methods for teaching invasive skills because of the superior learning effects of the student-on-student practice.

*I have taught nursing skills laboratory for decades. However, I still pray when I get to the injection practice. This is because there can be side effects and anatomical anomalies*.

(Participant A)

*There are many nursing skills lab instructors who supervise 25 students by themselves over their working hours, and I feel they are forcing themselves to make sacrifices for student safety*.

(Participant F)

#### 3.1.3. Conflict between Students’ Physical Safety and Patients’ Safety

The participants also mentioned the conflict between ensuring students’ physical safety and the safety of patients that students would care for as future nurses. If instructors began employing practice models to teach invasive procedures, this might cause future patient problems (e.g., high distrust) because nurses would not practice on actual humans until after graduation. Participants mentioned that they felt that the ethical guidelines were unclear regarding whether they should prioritize students’ physical safety or that of patients. Such conflict lies in the bipolar nature of human rights and respect for life. Before applying nursing skills to patients, nurses and nursing students need to be thoroughly experienced, namely by practicing as if was the actual clinical situation, using real human bodies. This can be considered as substantial in respecting patients’ rights and their lives. However, if we consider the same matter from the students’ stand point, it is difficult to practice nursing.

*It is true that the patients feel somewhat relieved when notified that the students have gone through hands-on lab exercises. However, I was often times confused in terms of ethics regarding whether student safety or patient safety should be prioritized*.

(Participant D)

### 3.2. Theme 2. Perceptions Related to Psychological Safety

#### 3.2.1. Students’ Feelings of Embarrassment Related to Psychological Safety

Instructors recognized that students felt embarrassment when exposing their bodies or engaging in physical contact during student-on-student practice. This embarrassment occurred during practice between students of the same gender as well as those of the opposite sex, especially when students did not want to reveal physical issues such as scars, wounds, or skin diseases.

*Students are naturally ashamed of physical exposure or contact. Even if a female student is paired with a female student for practice, they are ashamed about it*.

(Participant H)

#### 3.2.2. Lack of Knowledge Related to Psychological Safety

However, when the researcher asked questions about psychological safety, the participants asked for meaning as a reverse question and requested examples of psychological safety. They were able to state categories of psychological safety and shared their experience but did not think it was related to the safety problem and necessary to be reported.

*What is psychological safety*?

(Participant A)

*For example, in intramuscular injection, I supervised male students. It’s better for an older person to do it… younger instructors are likely to fell…a little discomfort to have male students. Anyway, I don’t think I’ve ever felt uncomfortable or anything like that*.

(Participant C)

*I think it can be shameful or embarrassing for the instructor to expose student’s bodies to practice nursing skills. If the instructor asked a student, “Can you do it for me?” It’s hard to say no when asked. Now that I think about it, that’s true. I didn’t recognize it then...*

(Participant G)

### 3.3. Theme 3. Inadequate Safety Reporting System

Participants mentioned the lack of clear definitions, scope, regulations, and reporting systems for accidents that occur in the classroom when teaching physically invasive procedures. In the past, instructors did not write incident reports if they were able to handle an accident themselves. However, participants now want safety regulations not only within the nursing department but also at the school level.

*The definition of a safety accident is ambiguous, so it is unclear whether an incident report should be used*.

(Participant A)

*There have been many cases of hematoma after the IV practice, but we didn’t report it as a safety accident*.

(Participant C)

## 4. Discussion

This study explored the experiences of instructors regarding the use of students as practice models in nursing skills laboratories. A student practicing nursing skill on other students is a technique widely used when teaching nursing skills in the Republic of Korea. Most nursing instructors reported that acting as a model for the demonstration was a burdensome experience for the students.

Instructors also mentioned a dilemma between the experimental learning of students and the need to keep them safe. In a previous study, 51.4% of instructors reported that students had experienced physical injuries in nursing skills laboratories [5]. These prevalence rates are not ignorable and align with instructors’ apparent fear of students experiencing physical injuries during nursing practice reported during this study. Faculties should pay high attention to signs or symptoms of physical unsafety. Before practicing nursing skills, faculties should emphasize and train students regarding safety strategies, such as avoiding recapping needles, wearing protective devices, and always replacing the sharps disposal container, etc.

Despite these risks, many nursing students gave instructors positive feedback on the use of the student-on-student practice. This finding aligns with research showing that practicing on the human body leads to greater student satisfaction than practicing on a manikin [6]. To help instructors with this challenge, reducing the student-to-faculty ratio, which would ensure supportive and careful supervision, and thereby increasing student safety, would be beneficial. Additionally, it might be appropriate to use nursing training equipment that more closely resembles a human than the equipment that is traditionally used in classrooms [20,21]. 

The instructors stated that they felt a dilemma between students’ and patients’ safety. If students had no chance to practice nursing skills on human bodies during nursing practice, they would first practice at the hospital on patients’ bodies. Therefore, nursing educators are concerned about balancing the physical safety of patients with that of nursing students. As nursing students who take part in nursing practice tend to be novices, it is unrealistic to expect that they can practice nursing skills on their classmates. Indeed, such an expectation can threaten student safety. Therefore, it may be sufficient to have students engage in supervised practice on a manikin at the outset [22]. Patient safety can be ensured by providing students with practice opportunities.

Instructors also mentioned that students often felt embarrassed when exposing their bodies to other classmates or when engaging in physical contact. Edmondson [23] proposed the concept of psychological safety, which refers to an individual’s perception of their learning environment as having no negative consequences (e.g., embarrassment or distress). Maintaining psychological safety can reduce learner anxiety and allow students to engage in the clinical setting, thereby reducing the number of mistakes they make in nursing skills performance and increasing students’ satisfaction with learning and desire to learn [24,25,26]. In this study, the embarrassment that students reportedly felt might have reduced their psychological safety, which could negatively affect their learning. However, instructors were less sensitive to recognizing this embarrassment as a problem of psychological safety and did not feel compelled to report it. Therefore, nursing instructors should strive to maintain academically safe environments to elicit psychological safety, such as preserving student privacy and ensuring students’ autonomy and independence [26,27]. In addition, students need to develop communication skills and speak up if any psychological safety issue occurs, without fear or embarrassment. When students felt safe, they were more fully engaged to learning experiences [26].

Finally, instructors were distressed about the inadequate safety reporting system for the nursing students. Currently, there are defined and well-developed management systems to ensure patient safety in nursing education; however, student safety and its scope have not been clearly defined, and there is no systematized incident reporting system [28]. Given these circumstances, instructors mentioned that they experienced considerable pressure and frustration when students had accidents during nursing practice. A previous study [5] indicated that informed consent might help make students fully aware of the physical and psychological risks of the student-on-student practice of such procedures. Students should be aware that they have the right to refuse to participate in these nursing procedures. Hence, developing well-organized student safety management systems in the future, including informed consent, a reporting system, and an accident management system, would be necessary to reduce the distress of nursing educators teaching nursing practice.

According to our findings, the Fundamentals of Nursing practice instructors face a dilemma between the positive effects of students practicing skills on each other and the impact of possible safety concerns. Policies aimed at protecting student safety in education on nursing skills and providing opportunities to practice nursing skills to understand what it feels like to receive clinical care should be considered and developed in the future.

Several strategies can be employed for student safety. First, a well-organized accident report system should be created. Second, a smaller student-to-instructor ratio during the Fundamentals of Nursing practice laboratory might decrease the occurrence of safety accidents through instructors’ close supervision. Third, the development of equipment that closely resembles human bodies would provide sufficient practice opportunities before student-to-student practice. Finally, before starting the practice, it is recommended to receive informed consent, including protecting the confidentiality and personal information or health history that the partner obtained during hands-on practice. Moreover, faculties need to acknowledge and share details step by step about what will happen during practicing nursing skills on students’ body to develop safer learning environment for nursing students.

Furthermore, nursing associations should raise concerns about student safety during practice and provide recommendations to instructors regarding how to promote student safety.

### Limitations

Our results should be interpreted in light of the limitations of this study design. First, the study outcomes may not represent the distress of all nursing instructors because the results were influenced by the interactions among the participants in the focus group interviews, and the sample size included only eight nursing instructors. In addition, this study focused on the instructors’ viewpoint; therefore, additional studies examining students’ perceptions and their distress regarding safety in nursing skills laboratories are needed. Finally, this study has limitations that can be applied to specific circumstances only when the students practice nursing skills using their bodies as a practice model. However, no instructors and nursing students can go through nursing education programs without confronting such issues along the way. Therefore, the current study will be of interest for the general nursing audience as well.

## 5. Conclusions

We presented the experience of nursing instructors regarding the use of students as practice models in nursing skills laboratories. Our findings indicate that instructors experienced a dilemma between the experimental learning of students and the need to keep students safe. Instructors and programs should implement safer methods of skill practice to maximize student learning. In addition, it is necessary to develop various human body training models that can ensure the physical and emotional safety of students. Furthermore, comprehensive safety management guidelines for laboratory practice must be developed; patient safety should not precede student safety. Therefore, nurse educators must contemplate the balance between risks and benefits when using nursing students as practice models in nursing skills laboratories.

## Figures and Tables

**Table 1:** General Characteristics of Participants.

	Gender	Age	Education	Experience of Teaching Nursing Skills	Number of Students in Class	Clinical Experiences
A	Female	62	Ph.D.	37 years	25	1 year
B	Female	48	Ph.D.	9 years	20–22	6 years
C	Female	61	Ph.D.	35 years	7–8	3 years
D	Female	49	Ph.D.	10 years	22–24	3 years
E	Female	45	Ph.D.	6 years	23–25	6 years
F	Female	43	Ph.D.	8 years	10–15	5 years
G	Female	48	Ph.D.	7 years	20	3 years
H	Female	58	Ph.D.	19 years	20–25	4 years

Ph.D. = Doctor of Philosophy.
